# Factors associated with non-adherence to insulin in patients with type 1 diabetes

**Published:** 2014

**Authors:** Musarrat Riaz, Abdul Basit, Asher Fawwad, Muhammad Yakoob Ahmedani, Zahara Ali Rizvi

**Affiliations:** 1 Musarrat Riaz, FCPS, Consultant Physician, Department of Medicine, Baqai Institute of Diabetology and Endocrinology, Baqai Medical University. Plot No. 1-2, II-B, Nazimabad No2, Karachi-74600, Pakistan.; 2 Abdul Basit, FRCP, Professor of Medicine, Department of Medicine, Baqai Institute of Diabetology and Endocrinology, Baqai Medical University. Plot No. 1-2, II-B, Nazimabad No2, Karachi-74600, Pakistan.; 3 Asher Fawwad, M.Phil, Assistant Professor, Research Department, Baqai Institute of Diabetology and Endocrinology, Baqai Medical University. Plot No. 1-2, II-B, Nazimabad No2, Karachi-74600, Pakistan.; 4 Muhammad Yakoob Ahmedani, FCPS, Professor of Medicine, Department of Medicine, Baqai Institute of Diabetology and Endocrinology, Baqai Medical University. Plot No. 1-2, II-B, Nazimabad No2, Karachi-74600, Pakistan.; 5 Zahara Ali Rizvi, MPhil (Student), Dept of Health Education & Health Promotion, BIHS, Mirpur, Dhaka, Bangladesh.

**Keywords:** Non-adherence, Patients with type 1 diabetes

## Abstract

***Objectives:*** To find out the various factors associated with non-adherence to diet, physical activity and insulin among patients with type 1 diabetes. (T1DM).

***Methods:*** This cross sectional study was conducted among T1DM subjects attending the Baqai Institute of Diabetology & Endocrinology (BIDE) and Diabetic Association of Pakistan (DAP), from July 2011 to June 2012.Clinical characteristics, anthropometric measurements, knowledge regarding type 1 diabetes along with adherence to dietary advice, physical activity and insulin were noted on a predesigned questionnaire and score was assigned to each question. Patients were categorized as adherent or non-adherent on the basis of scores obtained. Statistical Package for Social Sciences (SPSS) for windows version 17.0 was used to analyze the data.

***Results:*** A total of 194 patients (Male 94, Female 100), with mean age of 17.9± 6.4 years, mean duration of diabetes 5.37±4.96 years (38.1% >5 yrs, 61.9% <5 yrs) were included in the study. One hundred and fourteen (58.5%) patients were non adherent to dietary advice, 82(42.3%) non adherent to physical activity while 88.1% respondents were non adherent to their prescribed insulin regimen. Factors associated with non-compliance were family type, occupation & educational level of respondent’s parents, duration of T1DM, family history of diabetes, frequency of visits to diabetic clinic, knowledge regarding diabetes, lack of family support and fear of hypoglycemia.

***Conclusion:*** Non adherence to prescribed treatment regimen in patient with TIDM is quite high. There is need to design strategies to help patients and their family members understand their treatment regimen in order to improve their adherence.

## INTRODUCTION

Diabetes is a chronic disorder and the process of care of patients with diabetes is complex. It requires frequent self-monitoring of blood glucose, exercise, dietary modifications and administration of medications and/ or insulin.^1^^-^^3^ Non-adherence to prescribed treatment schedule continues to be a major problem the world over especially for medications in chronic diseases.^4^^,^^5^ The diabetes control and complication trial (DCCT) and other intervention studies^6^^-^^10^ demonstrated that achieving optimal glucose control through adherence to medications, exercise and diet prevents or minimize serious long term complications.^1^^-^^4^^,^^11^^-^^13^

Adherence has been defined as the “active, voluntary and collaborative involvement of the patients in a mutually acceptable course of behavior to produce a therapeutic result”.^14^ Non adherence to prescribed treatment regimen is common in patients with diabetes ranging from 23-77%, making optimal glycemic control difficult to achieve. Various factors have been identified contributing to non-adherence and include age, gender, disease duration, family factors, poor patients provider relationship, side effect of medication and financial constraints.^1^^-^^4^^,^^15^^,^^16^ There are many different methods of measuring adherence in type 1 Diabetes Mellitus (TIDM). The gold standard being electronic measurement, which in TIDM many include self-monitoring of blood glucose via glucometer and insulin usage via an insulin pump. Other measures include patient self report, structured interviews, and patient questionnaires.

Most of the studies regarding non-adherence to treatment were done in developed countries, where the health care delivery system is different from developing countries. Data on the predictors of non-adherence from developing countries, especially Pakistan is scarce. Better adherence will certainly translate in improved treatment efficacy, better intervention outcomes and reduction of cost of burden on health care. This study was therefore carried out among a sample of TIDM patients to determine the frequency and factors that are associated with non-adherence to prescribed diabetes treatment regimen.

## METHODS

This was a hospital based cross sectional study conducted among patients with type 1 diabetes attending Baqai Institute Of Diabetology & Endocrinology (BIDE), a tertiary care diabetes unit and Diabetic Association of Pakistan (DAP), a primary care diabetes center of Karachi Pakistan between Oct-2011 - June 2012. Formal approval of the study was obtained from Institutional Review Board of BIDE.

***Inclusion criteria: ***The main inclusion criteria were patients who were diagnosed and registered at BIDE and DAP as type 1 diabetes, willing to participate in the study and attending the diabetes clinics during the study period and were diagnosed before the age of 30 years.

***Exclusion criteria: ***Patients, who were unwilling to participate in the study, were very ill and those who were newly diagnosed (less than one month) with type 1 diabetes were excluded. Trained interviewers used a pre-tested structured questionnaire to obtain information on patient’s demographic characteristics and factors contributing to non-adherence to diabetes treatment.

***Data Collection: ***All the information regarding registered patients was saved in an electronic hospital data base called Health Management Information System (HMIS) against their code number. From the electronic data base, information about the age of onset of registered patients was taken and patients with T1DM were recruited accordingly. The newly registered patients were interviewed after two follow up visits within two months. Sample collection from DAP and BIDE were done on selected weekdays simultaneously.

Data were collected using preformed questionnaire. The questionnaire included 8 sections: Section A: Sociodemographic information, Section B: Clinical characteristics, Anthropometric measurements (Height in centimeters, and Weight in kilograms) Section C: Patterns of receiving Health care services, Section D: Knowledge regarding Diabetes, Section E: Behavioral factors, Section F: Adherences to Drug, Section G: Adherences to diet with 24 hour dietary recall interview, Section H: Adherences to physical activity advices.

- Knowledge scoring: Respondents who scored < 40 % were categorized as poor level of knowledge, respondents scoring between 40-60% were categorized as moderate level of knowledge, >60% score marked as good level of knowledge.
- Adherence to drug advices: Respondents were divided into two categories adherent and non-adherent based on their self-reported practices against recommendations. Adherent to medications were those respondents who scored 100% according to the recommendations given by physician. Respondents who scored less than 100% to recommended drug advices were categorized as non-adherent.
- Adherence to dietary advices- Respondents were divided into three categories; Those who scored >80% were considered “adherent” to dietary advice while those who scored between 60-80% were considered as “partial adherent” respondents scoring <60% were considered as “non-adherents
- Adherent to life style advices- respondents were categorized as adherent, partial adherent and non-adherent similar scoring criteria were used for assessment of adherence of life style advices.

***Statistical Analysis: ***Descriptive statistics were used for general description of study participants and to obtain the prevalence of non-adherence to diabetes treatment.

To assess the significant association of quantitative data independent t- test with p values was calculated. One way analysis of variance (ANOVA) was carried out to assess the significant difference between the groups followed by Post hoc: Bonferronni test for multiple comparisons to find out which group was significantly different with a total significance level of 5%.Data were presented by tables and graphs. All data were expressed as the mean ± SD and percentage. p value ≤ 0.05 was considered as significant.

## RESULTS

A total of 194 patients were included in the study. The average age of participants was 17.9±6.4 years. Majority of the respondents were females (51.5%) and about half of them (n=100, 51.5%) had primary education as the highest level of education attained. As regards educational status of respondents father, 91(46.9%) had higher secondary education, while most of the respondents mother n=170(87.6%) were housewives. One hundred and thirty five (69.6%) resided at a distance of less than fifteen kms from the hospital. One hundred and twenty (61.9%) respondents had less than 5 years duration of diabetes out of which 92(47.4%) patients visited the diabetes center twice or more in the last six months for follow up. The socio demographic characteristics of the patients are summarized in (Table-I).

**Fig.1:**
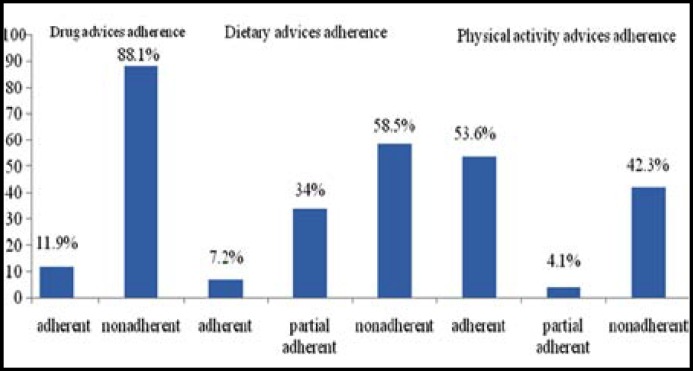
Distribution of respondents according to the proportion of adherence to drug, diet and physical activity advices (n=194).

The overall prevalence of non-adherence to prescribed treatment regimen among the respondents was 171(88.1%). One hundred and fourteen (58.5%) subjects were non adherent to dietary advices, while 82 (42.3%) patients were non adherent (Fig.1) to life style advices.

***a) Non adherence to prescribed treatment regimen: ***Factors found to be significantly associated with non-adherence were cost of insulin, occupation of respondent’s mother, family history of diabetes, poor understanding of prescription, irregularity of follow up, and fear of insulin. Age, gender, duration of diabetes and occupational status of respondent’s father was not significantly associated with non-compliance (Table-II).

***b) Non adherence to diet: ***As regards compliance to dietary advices, respondents with large family size (≥10 family members were more non-adherent (mean ±SD, 50.97±14.03, P≤0.05). Similarly patients having negative family history of diabetes were non-compliant (Mean±SD, 53.45±16.12, P≤0.05) as well as those patients who had least attendance to their diabetes clinic (<2) (Mean ± SD, 53.96±16.93) against those who had greater number of visits to diabetes clinic in last six months. Similarly those respondents who did not attend any diabetes educational program in the last 6-12 months were more non-adherent to dietary advices (Mean ± SD, 55.01±16.96).Table-III. On multiple regression analyses, total number of family members, family H/O Diabetes, and number of education programs attended in last 6-12 months were significantly (P≤0.05) associated with adherence to dietary advices after adjustment of other variables. Similarly positive correlation (r= 0.020) was found between knowledge score (independent) and total dietary adherence score (dependent)(Table-III).

***c) Non-Adherence to Physical Activity: ***Non adherence to physical activity was seen more in those respondents with positive family history of diabetes (Mean ±SD, 68.72±36.51) compared to those who had negative family history of diabetes (P≤ 0.05). Multiple regression analysis with adherence to physical activity advices as the dependent variable shows significant association with respondent’s maternal occupational status (P≤0.05), with their family history of diabetes (P≤0.05), their monthly family expenditure (P≤0.05), respondent’s total knowledge score (P≤0.05) and fear of hypoglycemia (P≤0.05) after adjustments of other variables (Table-IV).

## DISCUSSION

This study provides information regarding frequency of non-adherence to treatment among patients with type 1 diabetes and has explored factors associated with non-compliance to treatment.

**Table-I:** Distribution of the respondents according to the socio-demographic characteristics (n=194).

*Variables *	*Number (%)*
*Age *	
<18years	99(51)
≥18years	95(49)
*Sex*	
Male	94(48.5)
Female	100(51.5)
*Religion*	
Islam	189(97.4)
Others(Hinduism and Christianity)	5(2.6)
*Ethnicity*	
Punjabi	21(10.8)
Sindhi	32(16.5)
Muhajir	99(51)
Others (Balochis, Pashtuns, Memons)	42(21.6)
*Educational status of respondent*	
Illiterate-informal	24(12.4)
Pre-primary-secondary	100(51.5)
Higher secondary+	70(36.1)
*Educational status of respondent’s father*	
Illiterate-informal	52(26.8)
Primary-secondary	51(26.3)
Higher secondary+	91(46.9)
*Occupation of mother*	
House wife	170(87.6)
Others(Govt.services, teacher, business)	24(12.4)
*Residence type *	
Own house	134(69.1)
Rent house	52(26.8)
Govt. house	8(4.1)
*Type of family *	
Nuclear	115(59.3)
Joint	79(40.7)
*Family size*	
<10	162(83.5)
≥10	32(16.5)
*Distance from residence to hospital*	
**<**15km	135(69.6)
≥15km	59(30.4)

*n=number of subjects; results are expressed as number (%).*

**Table-II:** Factors associated with adherence to drug advices by independent sample t-test (n=194).

*Variables*	*Number * *(%)*	*Level of significance (p-value)*
*Age *		
<18 years	99(51.03%)	0.845
≥18 years	95(48.96%)	
*Sex*		
Male	94(48.45%)	0.328
Female	100(51.54%)	
*Occupation of respondent’s mother*		
Housewife	170(87.62%)	
Other professionals	24(12.37%)	0.180
(Govt. service/teacher/business)		
*Duration of T1DM*		
<5 years	120(61.85%)	0.825
≥5 years	74(38.14%)	
*Family history of T1DM*		
Yes	28(14.43%)	
No	166(85.56%)	0.751
*Family history of T2DM*		
Yes	108(55.67%)	
No	86(44.32%)	0.067
*Frequency of visits to diabetic clinics (in months)*		
<2	102(52.57%)	
≥2	92(47.42%)	0.20
*Briefing of prescription*		
Own language (Urdu)	175(90.20%)	0.102
Other language (English/Sindhi)	19(9.79%)	
*Fearness of insulin injection*		
Yes	145(74.74%)	0.09
No	49(25.25%)	
*Embarrassment of taking insulin*		
Yes	118(60.82%)	0.103
No	76(39.17%)	

*n=number of subjects; results are expressed as number (%)* *; p≤0.05 was taken as level of significance.*

**Table-III:** Factors associated with adherence to dietary advices by independent sample t-test (n=194).

*Variables*	*Number* *(%)*		*Level of significance* *(p-value)*
*Family type *			
Nuclear	115(59.27%)	0.417	
Joint	79(40.725)		
*Total no. of family members*			
<10	162(83.50%)		
≥10	32(16.49%)	0.05*	
*Family history of T1DM *			
Yes	28(14.43%)	0.27	
No	166(85.56%)		
*Family history of T2DM*			
Yes	108(55.67%)	0.04*	
No	86(44.32%)		
*Diagnosis of T1DM by*			
Physicians/diabetologists	142(73.19%)	0.135	
Others(internist/technician)	52(26.80%)		
*Frequency of visits to diabetic clinics(in months) *			
<2	102(52.57%)	0.05*	
≥2	92(47.42%)		

*n=number of subjects; results are expressed as number (%)* *; p≤0.05 was taken as level of significance, *

* *=significant*

**Table-IV:** Factors associated with adherence to physical activity advices by independent sample

*Variables*	*Number* *(%)*	*Level of significance* *(p-value)*
*Occupation of respondent’s mother*		
Housewife	170(87.62%)	0.02*
Other professionals (Govt. service/teacher/business)	24(12.37%)	
*Family type *		
Nuclear	115(59.27%)	0.09
Joint	79(40.72%)	
*Duration of T1DM*		
<5 years	120(61.85%)	0.30
≥5 years	74(38.14%)	
*Family history of T1DM*		
Yes	28(14.43%)	0.23
No	166(85.56%)	
*Family history of T2DM*		
Yes	108(55.67%)	0.02*
No	86(44.32%)	
*Frequency of visits to diabetic clinic*		
<2	102(52.57%)	0.39
≥2	92(47.42%)	
*Fearness of hypoglycemia*		
Yes	122(62.88%)	0.14
No	72(37.11%)	

*n=number of subjects; results are expressed as number (%)* *; p≤0.05 was taken as level of significance* *,*

* *=significant*

Majority of the respondents in this study were non adherent to prescribed medications. This level of non-adherence is quite high. Similar finding was observed in the study done in Saudi Arabia in which 79% of the patients were non-compliant to insulin regimen.^17^ A contrasting result was found in a study done in Uganda where only 28.9% patients were non-adherent to diabetes drug treatment due to non-availability of medical care service.^18^ Although our study populations were also under free consultation and insulin was available free of cost still a large proportion of non-adherence to drug treatment indicates that some of the socio demographic and service related factors are influencing the patient compliance.

Non-adherence is likely to increase the complication of diabetes which will lead to increased cost of health care due to increase morbidity. The risk of non-adherence in our study was increased among patients who did not understand their prescription which cause a negative impact on patients understanding to their prescribed drug regimen by their physicians. This is comparable to the finding of a study done in Uganda which showed that non-adherence to drugs was significantly related with understanding of the patients about their treatment (p=0.0.01).^18^

Maternal education was found to be significantly associated with non compliance. Similar findings were observed in a study done in Tanzania which showed that education of the caregiver is important to ensure adherence to the multiple diabetes related tasks among T1DM patients.^19^

In the current study more than half of the respondents (58.5%) were non adherent to dietary recommendations. Similar findings were observed in a qualitative study done in Karachi, Pakistan in which most of the participants found difficulty in adhering to their dietary advices.^20^ Similar findings are observed in studies from Finland, USA and Norway which stated that many youth with T1DM did not adhere to daily recommendation for dietary advices.^21^^-^^23^ Likewise, in studies done in Cuba and in the United states, 70-75% of study participants reported non adherence to dietary recommendation.^24^^,^^25^ A study done in India also stated that only 37% of the respondents were following dietary prescriptions regularly.^26^

Respondents with large family size were non-adherent to dietary advices than those with small family household. Probably in extended families where members with different needs and requirements live together specific diabetes related dietary requirements are not met.

Our finding showed that attendance to diabetes clinics as well as diabetes education programs reduce the non adherence to their dietary recommendation. This result correspond with the study finding in Alexandria, Egypt which observed a higher proportion of adherence to treatment among those who received health education.^27^ Similarly in a study from Uganda it was reported that non adherence was low among those respondents who visited their health worker and had attended health education session in the last 6 months.^28^ The diabetes dietary care regimen is complex, generally unpleasant, involving many imposition and restrictions, therefore dietitians need to consider demographic characteristics to tailor education sessions with patients to increase their understanding of dietary advices.

Physical activity play a vital role in the self management of T1DM and exercise is the best predictor of maintaining weight, and, independent of weight loss, it decreases insulin resistance. In assessing the non adherence proportion, nearly half (42.3%) of the participant were not following their physical activity advices. This is similar to finding of the study done in Islamabad, where 73.7% of the patients were non-compliant with their exercise regimen although quiet high percentage compared to our study.^29^

Lack of parental support result in non adherence to physical activity advice as per our study finding. This association is similar to the finding of the study done in Michigan; USA that showed parental support of exercise activity is related to higher rates of physical activity among youth with T1DM.The support includes encouragement and parents participation in the exercise activity.^30^

Lack of knowledge regarding importance of physical activity and life style modification in the overall management of diabetes and increased family expenditure showed a significant association with physical activity in our study. It indicates that respondents with high expenses are non adherence than those with low monthly expenditures. This may be due to sedentary life style among the affluent.

Fear of hypoglycemia is one of the factors for non adherence to physical activity. In the present study respondents with fear of hypoglycemia were more prone to not adhering to their physical activity advices. This is consistent with the finding of the study done is Scotland which stated that main reason for inactivity is due to fear of hypoglycemia.^31^

## CONCLUSION

Non adherence to prescribed treatment regimen in patient with TIDM is quite high. There is need to design strategies to help patients and their family members understand their treatment regimen including dietary and physical activity advices in order to improve their adherence. Multidisciplinary approach consisting of physicians, dietician and diabetes educator can be of great help in achieving this goal.

## Author Contributions:

***AB, AF, MYA, ZAR: ***Substantial contributions to conception and design, or analysis and interpretation of data.

***MR, AB, MYA:*** Drafting the article or revising it critically for important intellectual content.

***MR, AB, AF, MYA:*** Final approval of the version to be published.

## Notes

***Declaration of Competing Interests: ***Nothing to declare.
